# FINDING COMMON GROUND: IMPROVING RESEARCHER-PRACTITIONER COLLABORATION IN ADULT MALTREATMENT RESEARCH

**DOI:** 10.1093/geroni/igad104.1569

**Published:** 2023-12-21

**Authors:** Marguerite DeLiema, Kathleen Wilber

## Abstract

The work of adult protection is inherently multidisciplinary–involving health care professions, social services agencies, law enforcement, criminal and civil justice systems, and the broader aging services network. Researchers seeking to develop and evaluate screening tools and interventions for elder abuse and neglect must cultivate partnerships with state and local agencies that are responsible for adult protection. In this symposium, early career researchers will share the unique challenges they have faced when collaborating with adult protective services (APS), healthcare organizations, and other state agencies on research to improve adult maltreatment outcomes. First, Reynolds and colleagues will summarize three collaborative projects involving a large healthcare system in Texas and APS agencies in Oklahoma. They will share how the research team adapted protocols to pandemic-related disruptions to data collection, and how they created new systems to standardize documentation. Liu interviewed state agencies in Montana and analyzed transcripts using thematic analysis. She will share the challenges and strategies that surfaced and relate to improving state-level collaboration in addressing the needs of mistreatment victims and survivors. DeLiema will present on planning for and navigating administrative barriers to cross-sector collaborations, including contracts and legal agreements that are part and parcel to university-government research partnerships. Last, Burnes will introduce the RISE project and share how he and his colleagues managed expectations to co-create a new outcomes measurement tool with adult protection workers. Reaching consensus necessitated honesty, openness, humor, and patience, but ultimately strengthened the collaboration to yield a more valid outcomes measurement assessment.

